# Aircraft lavatory wastewater surveillance for movement of antimicrobial resistance genes: a proof-of-concept study

**DOI:** 10.1128/spectrum.00569-25

**Published:** 2025-05-28

**Authors:** Yawen Liu, Wendy J. M. Smith, Metasebia Gebrewold, Nicholas J. Ashbolt, Ishi Keenum, Stuart L. Simpson, Xinhong Wang, Warish Ahmed

**Affiliations:** 1 State Key Laboratory of Marine Environmental Science, College of the Environment & Ecology, Xiamen University 12466, Xiamen, Fujian, China; 2 CSIRO Environment, Ecosciences Precinct 117158 , Dutton Park, Queensland, Australia; 3 Future Industries Institute, Cooperative Research Centre for Solving Antimicrobial Resistance in Agribusiness, Foods and Environments, Adelaide University 613394, Mawson Lakes, South Australia, Australia; 4 Department of Civil, Environmental, and Geospatial Engineering, Michigan Technological University 3968, Houghton, Michigan, USA; Ocean University of China, Qingdao, China

**Keywords:** aircraft wastewater surveillance, AMR, ESKAPE pathogens, global transmission, ARGs, qPCR

## Abstract

Long-haul flight aircraft wastewater may serve as a representative microbial footprint, often of mixed country origin, offering valuable insight into the movement of pathogens and antimicrobial resistance (AMR) on a global scale. Herein, we present a proof-of-concept for aircraft-based surveillance of AMR by investigating lavatory wastewater samples from 44 repatriation flights to Australia departing from nine countries. Profiles of pathogens including ESKAPE pathogens (*Salmonella* spp., *Mycobacterium* spp., *Enterococcus faecium*, *Staphylococcus aureus, Klebsiella pneumoniae*, *Acinetobacter baumannii*, and *Pseudomonas aeruginosa*) and antibiotic resistance genes (ARGs) (*aph(3’)-IIIa*, *bla_NDM-1_, bla_CTX_M-1_, bla_KPC_, ermB, qnrS, sul1, tetM,* and *vanA*) were investigated along with traditional fecal indicator bacteria (*Escherichia coli* and *Enterococcus* spp.) and fecal/urine marker genes (*Bacteroides* HF183, *Carjivirus*, human polyomavirus, and a cryptic plasmid pBI143) using quantitative PCR (qPCR). Two fecal indicator bacteria (FIB) and four human fecal/urine marker genes were detected in all aircraft wastewater samples. Detection rates for ESKAPE pathogens ranged from 6.8% (*S. aureus*) to 84.1% (*K. pneumoniae*). Of all ARG targets, *aph(3’)-IIIa*, *ermB*, *qnrS*, *sul1,* and *tetM* were detected in all wastewater samples, whereas *bla_KPC_* and *vanA* were not detected in any of the samples. Results reflected geographic differences in ARG abundance originating from departure countries/continents and suggested a potential risk of importing ARGs that might be rare in local wastewater systems. The loss of nucleic acid targets was less than 10% over a 24 h incubation in the presence of disinfectants, suggesting that nucleic acids are resilient enough to persist in aircraft wastewater over the maximum duration of a flight.

**IMPORTANCE:**

In the context of international connectedness, aircraft-based wastewater surveillance should be viewed as a beyond-national tool to enhance global AMR management and foster international cooperation.

## INTRODUCTION

The global transmission of infectious diseases has been expedited by intercontinental flights (1). Various human pathogens, including *Mycobacterium tuberculosis* (*M. tuberculosis*) (2), influenza A virus (3, 4), multi-drug-resistant bacteria (5), and SARS-CoV-2 (6), are all known to have been spread because of air travel, with the potential to initiate or propagate infectious diseases outbreaks worldwide (7).

To reduce infectious viral disease transmission, multi-layered control strategies have been trialed, including travel restrictions, individual passenger screening, and quarantine (8, 9). During the coronavirus disease 2019 (COVID-19) pandemic, analyses of aircraft lavatory wastewater from international flights proved to be useful for monitoring the importation of SARS-CoV-2 (6, 10–13). Analyzing wastewater samples from aircraft could provide a non-invasive and efficient tool to support the established public health risk management efforts compared to extensive clinical testing of individual passengers (7, 14). Passengers infected with pathogens are likely to shed them in their stool and body fluids (i.e., urine, saliva, sputum, etc.) when using the onboard lavatories (12, 15). A recent study by Knight et al. (16) highlighted aircraft wastewater as a potential route for cross-border AMR transmission and a valuable tool for global AMR surveillance (16).

Antibiotic-resistant bacteria (ARB) are among the top global public health threats, responsible for an estimated 1.27 million global deaths in 2019 (17) and predicted to rise cumulatively from 2025 to 2050 to result in more than 39 million deaths that are directly attributable to antimicrobial resistance (AMR) (18). Increasing international mobility of people has significantly contributed to the global spread of antimicrobial resistance (AMR), as travelers to areas with high AMR prevalence are likely to be exposed to ARB and return to their countries carrying these bacteria (5). For example, a study from an international airport found that more than 90% of the *Escherichia coli* (*E. coli*) isolates from aircraft wastewater were resistant to at least one commonly used antibiotic, compared to 45%–60% for local untreated wastewater (19). Among AMR infections, *E. coli*, along with a group of ESKAPE pathogens (*Enterococcus faecium*, *Staphylococcus aureus*, *Klebsiella pneumoniae*, *Acinetobacter baumannii*, *Pseudomonas aeruginosa,* and *Enterobacter* spp.), has been identified as critical multidrug-resistant bacteria that pose major therapeutic challenges worldwide (20, 21). Evidence from international travelers also revealed high acquisition of ARB (e.g., extended-spectrum β-lactamase-producing *Enterobacteriaceae*) and antibiotic resistance genes (ARGs) encoding resistance to sulfonamide, trimethoprim, and beta-lactams in the gut microbiome after their return (22, 23).

Given the escalating scale of international travel, the global transmission of ARB with novel ARGs to the destination region is expected to accelerate due to high cross-border mobility, as seen previously with NDM carbapenemases (24). This extension to transmission will pose a great burden on modern medicine in the global combat against bacterial infections (17). In the effort to manage AMR, aircraft wastewater surveillance presents a complimentary approach for detecting emerging ARBs and ARGs, track their origins, monitor their global spread, and thus provide disease intelligence to support local responses and to enhance global health emergency and coverage (14).

To date, only three studies have conducted detailed AMR analysis of lavatory waste from international flights, using high-throughput quantitative PCR (qPCR) and sequencing to detect multiple human pathogens and ARGs (16, 25, 26). Herein, we demonstrate the feasibility and applicability of aircraft-based wastewater surveillance for AMR by (i) investigating and comparing the abundance of fecal indicator bacteria (FIB), several fecal/urine maker genes, ESKAPE pathogens, and clinically important ARGs in aircraft lavatory wastewater samples collected from 44 repatriation flights to Australia and (ii) evaluating the effect of disinfectants on the persistence of nucleic acids to support aircraft-based wastewater surveillance. Our preliminary findings provide data required to enhance the global surveillance network for mapping AMR transmission and potential early warning of emerging threats to public health.

## MATERIALS AND METHODS

### Wastewater sampling from the aircraft lavatory

Aggregated wastewater samples (500 mL to 1 L) were collected from the wastewater exiting lavatories of 44 repatriation flights landing at Darwin International Airport (DRW), Northern Territory, Australia, between 8/NOV/2020 and 7/SEP/2021 (Table S1). These samples were archived at our laboratory at −20°C and previously used for SARS-CoV-2 screening (12).

Wastewater samples were collected in Darwin, Australia, from international flights that had departed from different countries, including 18 flights originating from India, 14 flights from the United Kingdom (UK), six flights from Germany, and single flights from France, UAE, Turkey, South Africa, Japan, and Indonesia. All these flights were long-haul, lasting more than 6 h, except for one flight originating from Indonesia, which had a duration of approximately 2.5 h. Each flight accommodated approximately 94–195 passengers. These flights were selected with the expectation that the repatriation passengers would consume food on one or more occasions, were more likely to use the lavatory during the flight, and were likely to defecate on board (13). Wastewater was sampled using a specifically designed sample trap capable of retaining an aliquot of bulk wastewater sample exiting the aircraft lavatory before entering a waste service truck system (https://zenodo.org/records/7834950). More details about the wastewater collection process could be found in our previous study (13).

### Decay of nucleic acid targets from the lavatory disinfectant

The decay of nucleic acids in wastewater during flight in the presence of disinfectants and at a constant temperature (~15°C) was investigated by setting up laboratory microcosms designed to mimic an aircraft lavatory environment. Wastewater samples used for microcosms were composite fresh (i.e., used immediately after collection) wastewater samples collected from the influent of a WWTP. The urban wastewater samples were used instead of aircraft wastewater due to a lack of access to fresh aircraft wastewater samples. While the bacterial profiles of urban and aircraft wastewater may vary in diversity, both originate primarily from the human gut, making urban wastewater a suitable proxy for this study. In addition, the bacterial taxa selected for assessing nucleic acid decay are highly abundant and common in both wastewater sources, ensuring the reliability of the results. The composite wastewater sample was then mixed with the Novirusac Gel Bulk (Aero Defence Pty. Ltd, Southport, Queensland, Australia), a hospital grade disinfectant liquid which is commonly dosed into the Australian aircraft’s tank prior to departure, at three different ratios (0.1%, 1%, and 10% vol/vol).

The Novirusac Gel Bulk is comprised of hexylene glycol, benzalkonium chloride, didecylmethylammonium propionate ethoxylated, N-(3-aminopropyl)-N-dodecyl-1,3-propanediamine, ethanolamine, and water. The 1% dilution ratio of Novirusac Gel is recommended for routine use in recirculating aircraft lavatories (https://www.itwpf.com.au/products/applied-novirusac-gel-bulk-aircraft-lavatory-treatment-25l). The 0.1% and 10% dilution ratios were selected to account for both the dilution effects of target concentrations and the worst-case scenarios of target degradation in aircraft wastewater samples. Microcosms (5 mL capped sterile Eppendorf tubes) were then aliquoted in triplicate from three mixtures of wastewater and disinfectants, each at different dilution ratios, and incubated at 15°C in a water bath fitted with a thermostat to maintain a constant temperature. A control microcosm, aliquoted from the composite wastewater sample without the addition of any disinfectant, was incubated alongside the other microcosms.

All microcosms underwent incubation for up to 24 h, representing the maximum flight duration. The control microcosm, along with the biological triplicate microcosms for each dilution ratio group, was removed from the water bath at 0 h (the start of incubation), 3 h, 6 h, 9 h, 12 h, 15 h, and 24 h, and then stored at 4°C overnight. Nucleic acid was extracted from wastewater samples on the following day.

### Aircraft lavatory sample preparation and nucleic acid extraction

Aircraft lavatory wastewater samples were thawed at 4°C overnight. Both microcosms and aircraft wastewater samples were processed following the protocol detailed in Smith et al. (27). This protocol had been demonstrated to facilitate sensitive detection and quantification of ARGs in aircraft wastewater samples (27). Briefly, the 0.2 mL aliquots of each aircraft wastewater sample were centrifuged at 21,000 *× g* for 3 min, and the supernatant was discarded. The resulting pellets were subjected to nucleic acid extraction using the DNeasy Blood and Tissue kit (Cat. No. 69506) (Qiagen, Hilden, Germany). Each pellet was resuspended in 180 µL of ATL buffer, then 20 µL of proteinase K was added. The mixture was thoroughly vortexed and then incubated at 56°C for 60 min. After incubation, 200 µL of AL buffer was added and incubated at 56°C for 10 min. Finally, 200 µL of ethanol was added, and the resulting solution was loaded onto the spin columns, transferred to a rotor adapter on the QIAcube Connect platform, and processed following the manufacturer’s instructions. Nucleic acid was extracted and eluted in 200 µL of RNase-free water for aircraft wastewater and microcosm samples and stored at −20°C before qPCR analysis.

### qPCR assays

For aircraft wastewater nucleic acid samples, qPCR assays were used to quantify the bacteria 16S rRNA gene (28). Two FIB, *E. coli* 23S rRNA and *Enterococcus* spp. (ENT) 23S rRNA genes (29, 30), along with four human wastewater-associated marker genes, were targeted to provide evidence of onboard lavatory usage. These markers include *Bacteroides* HF183 (31), *Carjivirus* (formerly known as crAssphage) (32), human polyomavirus (HPyV) (33), and a “cryptic” plasmid named pBI143, which is reported to be abundant in urban wastewater and has been identified as having potential application for microbial source tracking (MST) (24, 25). In addition, species-specific functional genes were selected to quantify the *Mycobacterium* spp. 23S rRNA (34) and *Salmonella* spp. *ttr* locus (35), which had been reported as infections transmitted on commercial airlines (2).

The ESKAPE pathogens, identified as critical multidrug-resistant bacteria, were screened, encompassing *Enterococcus faecium* (*E. faecium* ddl gene) (36), *Staphylococcus aureus* (*S. aureus* nuc gene) (37), *Klebsiella pneumoniae* (*K. pneumoniae* khe gene) (38), *Acinetobacter baumannii* (*A. baumannii* bap gene) (39), and *Pseudomonas aeruginosa* (*P. aeruginosa* gyrB gene) (40). ARGs indicative of anthropogenic and clinically relevant sources were screened, including *aph(3′)-IIIa* (encoding an aminoglycoside-modifying enzyme) (41), *bla_NDM-1_* (encoding the New Delhi metallo-β-lactamase-1 enzyme) (42), *bla_CTX_M-1_* (encoding extended-spectrum β-lactamase enzyme) (43), *bla_KPC_* (conferring resistance to carbapenems in *K. pneumoniae*) (44), *ermB* (conferring resistance to erythromycin) (45), *qnrS* (conferring resistance to fluoroquinolone) (46), *sul1* (conferring resistance to sulfonamides by encoding dihydropteroate synthases) (47), *tetM* (conferring resistance to tetracyclines by encoding a ribosomal protection mechanism) (48), and *vanA* (conferring resistance to vancomycin) (49). The decay of nucleic acids in microcosms was analyzed using three qPCR assays targeting the 23S rRNA gene of ENT (representing the FIB), pBI143 (representing a human fecal marker), and the *tetM* ARG.

A series of qPCR standards (ranging from 3 to 3 × 10^6^ gene copies (GC)/reaction) prepared from synthetic DNA in plasmid cloning vectors or gBlocks gene fragments (Integrated DNA Technologies, Coralville, IA, USA) were used to quantify target genes and as qPCR positive controls. The qPCR primers, probes, and cycling parameters for all assays were provided in Table S2. All qPCR amplifications were performed in 20 µL reaction mixtures using 2 × QuantiNova Probe PCR Master Mix (Qiagen). The qPCR mixtures contained 10 µL of Master Mix, 200 to 1,000 nM of forward primer, 200 to 1,000 nM of reverse primer, 100 to 500 nM of probe, and 3 µL of template nucleic acid. All qPCR experiments were performed in triplicate using a Bio-Rad CFX96 thermal cycler (Bio-Rad, California, USA). Triplicate negative controls were included in each qPCR run. The threshold and baseline were all adjusted to the same values, ranging from 40 to 100 RFU for each qPCR assay.

### Quality assurance/controls

Quality assurance/control metrics, including amplification efficiencies (*E*), correlation coefficient (*r*^2^), and y-intercepts, are provided in Table S3 based on the Minimum Information of Publication of Quantitative Real-Time PCR Experiments (MIQE) guidelines (50). The assay limit of detection (ALOD) was defined as the minimum copy number with a 95% probability of detection for qPCR assays (51).

A Sketa22 real-time PCR assay was applied to determine PCR inhibition in extracted nucleic acid samples by seeding a known copy number (10^4^) of *Oncorhynchus keta* (*O. keta*) (29). The reference quantification cycle (Cq) value was determined from triplicate qPCRs containing only the positive control and compared with the Cq values obtained from all aircraft wastewater nucleic acid samples. Samples were considered to have no PCR inhibition when the Cq values of aircraft wastewater nucleic acid samples were within 2 Cq values of the reference Cq value (6). Nucleic acid extraction and qPCR setup were performed in separate laboratories to minimize contamination introduced in experiments. Filter blank and reagent blank were included in the processing of all aircraft wastewater samples.

### Statistical analysis

Aircraft wastewater samples were categorized as being positive for each target if amplification was observed within 40 cycles in at least one out of three qPCR replicates. Targets were categorized as non-detectable when no amplification was observed within 40 cycles in any of the qPCR replicates. Samples were categorized as quantifiable if amplifications were detected in at least two out of three qPCR replicates, and the Cq values were above the ALOD for each target. The concentrations of quantifiable aircraft wastewater samples were log_10_-transformed and expressed as log_10_ GC/L.

Data analyses were performed and visualized in R 4.3.2 (52) and GraphPad Prism Version 8.3.1 (GraphPad Software, La Jolla, CA). Concentrations of FIB, human fecal/urine marker genes, pathogens, and relative abundances of ARGs in samples from different countries (India, UK, and Germany) were compared using the Welch ANOVA or Kruskal-Wallis test, with the *P* values adjusted for multiple comparisons via Dunn’s test. The Mann-Whitney test was applied to assess statistical differences in the concentrations or relative abundances of targets in aggregated samples from different continents (Asia vs Europe).

The relative abundances of ARGs were calculated by dividing their concentrations by 16S rRNA GC or by GC of several fecal/urine markers (ENT 23 rRNA gene, HF183, pBI143, and *Carjivirus*), where each was used separately as the normalization factor. The effectiveness of these normalization factors was then assessed using principal component analysis (PCA) to compare the explained variance ratios of the first two principal components for each factor. *Carjivirus* was then used for normalization, allowing for the comparison of the prevalence of ARGs across flights. Density plots showing the relative abundance of three pathogens and nine ARGs were used to analyze the distributions of ARGs across flights departing from different countries or continents.

The correlations among FIB, human fecal/urine markers, bacteria 16S rRNA gene, pathogens, and ARG targets were visualized via a correlation matrix (Spearman’s rank-order correlation). In addition, correlation analysis was performed to assess whether the aggregated concentrations of each human fecal/urine marker in wastewater samples (*n* = 44) were associated with the number of people onboard. All data were tested for normality and homogeneity of variances before statistical analysis. *P*-values were marked with asterisks according to the usual convention, where * indicates *P* < 0.05, ** denotes *P* < 0.01, and *** corresponds to *P* < 0.001.

## RESULTS

### qPCR performance characteristics and PCR inhibition

For all qPCR assays, the correlation coefficients (*r*^2^) of qPCR standard curves were within the range between 0.994 and 1, and the qPCR efficiencies were within the range between 90% and 110%. The ALOD for each assay ranged from 4 GC/reaction to 10 GC/reaction. All aircraft wastewater nucleic acid samples were within the 2-Cq values of the reference Cq value, suggesting the likely absence of PCR inhibition in all nucleic acid samples.

### The impact of disinfectants on the decay of nucleic acids

**Fig 1:**
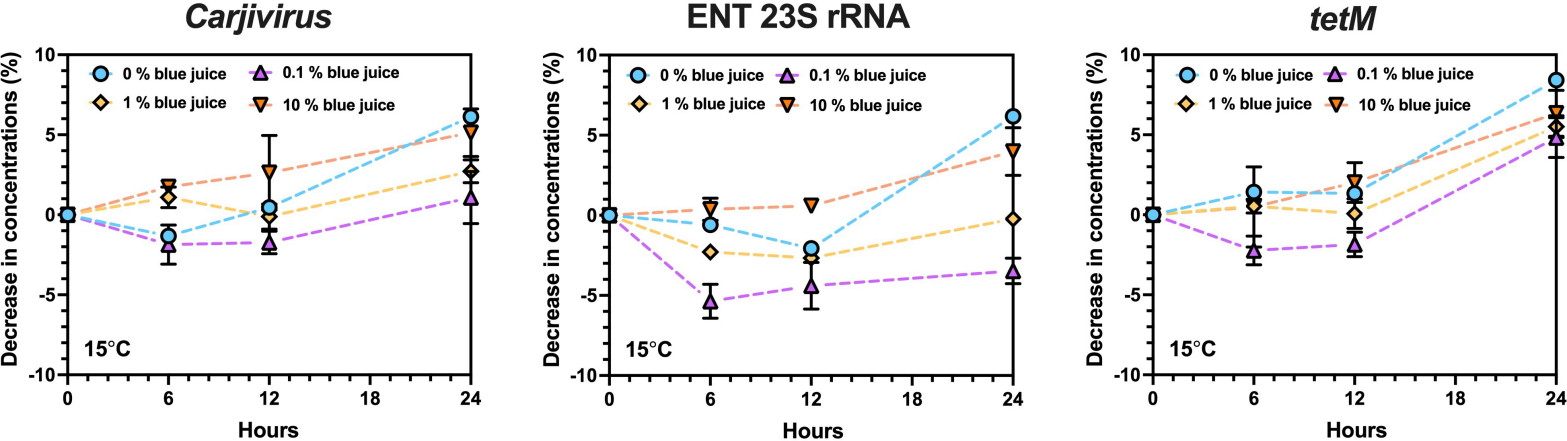
Percentage of decrease in concentrations of selected targets in the presence of disinfectant during the 24 h incubation

The nucleic acid of all endogenous targets was detected over the 24 h incubation at 15°C (Fig. 1). After the 24 h incubation, the percentage decrease in concentrations of ENT 23S rRNA gene, *Carjivirus,* and *tetM* in control microcosm sample was 6.2%, 6.1%, and 8.4%, respectively. For microcosm samples at 0.1%, 1%, and 10% dilution ratios, the mean percentages of concentration loss (%) increased from −3.5% to 4.0% for ENT 23S rRNA gene, 1.1% to 5.1% for *Carjivirus,* and 4.8% to 6.3% for *tetM*.

### Prevalence of human fecal/urine markers in aircraft wastewater samples

**Fig 2:**
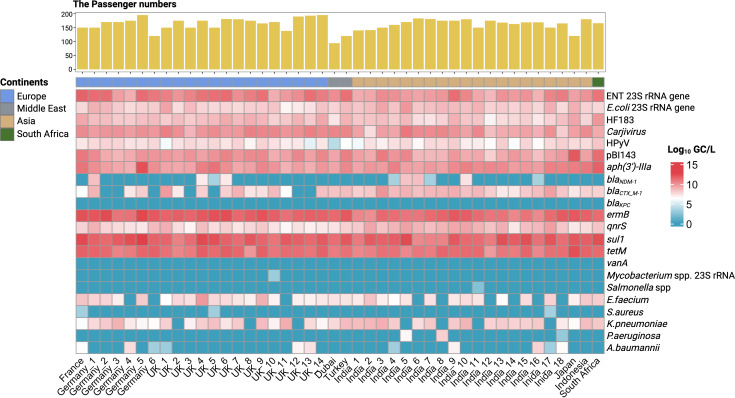
Prevalence of FIBs, human fecal/urine markers, pathogens, and ARGs in lavatory samples across flights.

**Fig 3:**
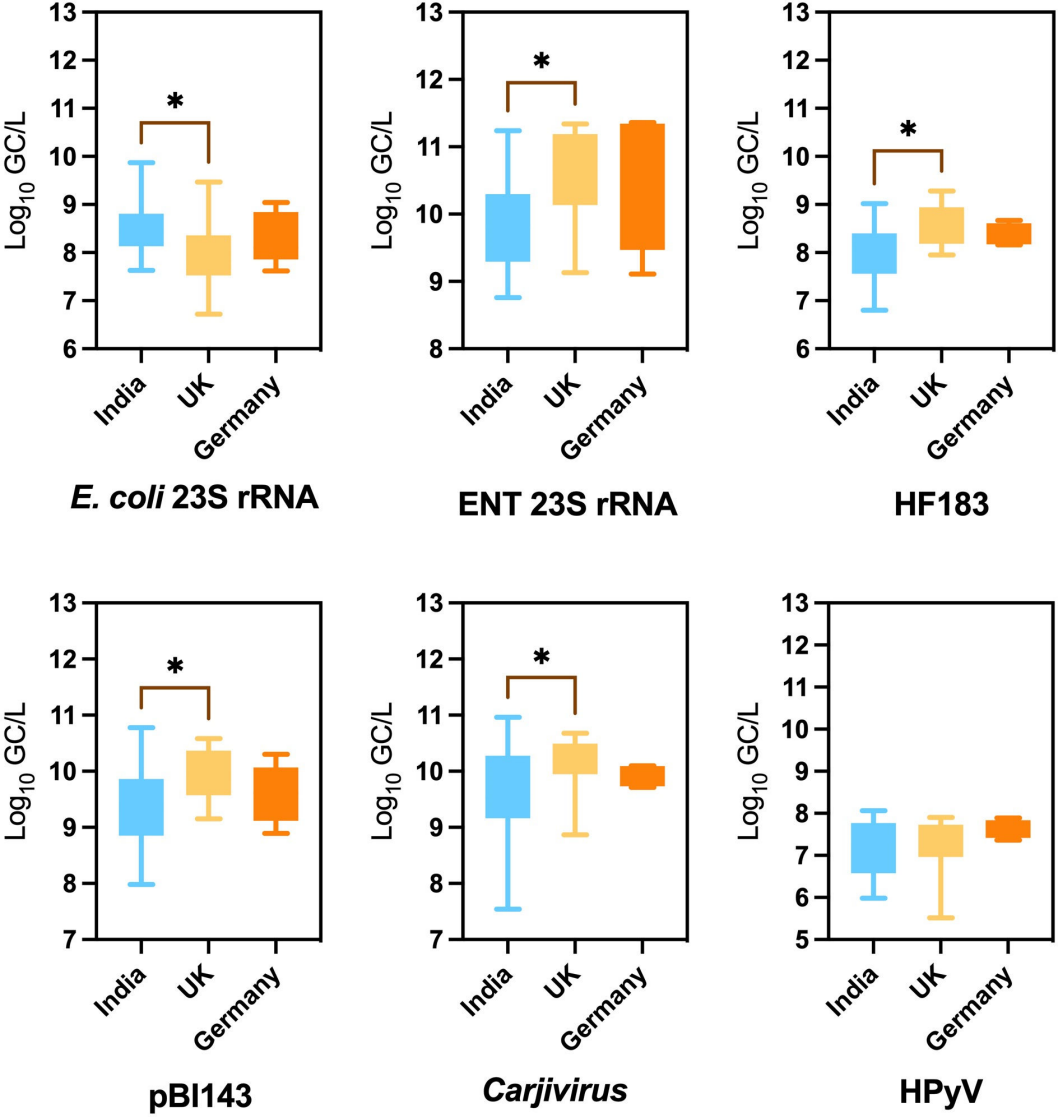
Concentrations of six human fecal/urine markers in flights differentiated by countries (* indicates *P* < 0.05).

Two FIB, the *E. coli* 23S rRNA and ENT 23S rRNA genes and four human fecal/urine marker genes HF183, pBI143, *Carjivirus,* and HPyV were detected in all aircraft wastewater samples (*n* = 44) (Fig. 2). The concentrations of *E. coli* 23S rRNA gene, ENT 23S rRNA gene, HF183, pBI143, *Carjivirus,* and HPyV ranged from 6.72 to 9.87 log_10_ GC/L, 8.76 to 11.7 log_10_ GC/L, 6.80 to 9.89 log_10_ GC/L, 7.98 to 11.67 log_10_ GC/L, 7.54 to 11.0 log_10_ GC/L, and 4.06 to 8.34 log_10_ GC/L, respectively (Table S4). One-way ANOVA revealed significant differences in concentrations of *E. coli* 23S rRNA gene, ENT 23S rRNA gene, HF183, pBI143, and *Carjivirus* among flights from India, UK, and Germany (*P* < 0.05). Significantly lower concentrations of those five markers in aircraft wastewater samples were observed in flights from India compared to those from the UK (*P* < 0.05), except for *E. coli*, which was significantly higher in flights from India than in those from the UK (*P* < 0.05) (Fig. 3). Similar patterns were found for aircraft wastewater samples when differentiated by the continent of departure (Fig. S1). Wastewater samples in flights from Asia exhibited significantly lower concentrations of ENT 23S rRNA gene (*P* < 0.01), HF183 (*P* < 0.01), pBI143 (*P* < 0.05), and *Carjivirus* (*P* < 0.05) than flights from Europe. Concentrations of *E. coli* 23S rRNA gene were significantly higher in flights from Asia compared to those from Europe (*P* < 0.05). Significant correlation was observed for the concentrations of HPyV with the number of passengers (*P* < 0.05, *r* = 0.33) (Fig. S2).

### Profiles of ARGs and pathogens in aircraft wastewater samples differentiated by the countries/continents of departure

Concentrations of putative pathogens varied from a median of 0 to more than a million GC/L in aircraft wastewater samples. Potentially pathogenic *Mycobacterium* spp. (3.09 log_10_ GC/L) and *Salmonella* spp. (2.07 log_10_ GC/L) were only detected in one flight from the UK and India, respectively. The detection rates for five ESKAPE-grouped pathogens were 79.6% for *E. faecium* (0–9.82 log_10_ GC/L), 6.8% for *S. aureus* (0–3.40 log_10_ GC/L), 84.1% for *K. pneumoniae* (0–9.15 log_10_ GC/L), 6.8% for *P. aeruginosa* (0–7.98 log_10_ GC/L), and 25.0% for *A. baumannii* (0–7.88 log_10_ GC/L) (Table S5). No significant difference was observed for concentrations of these five ESKAPE pathogens in flights departing from different countries or continents.

Of all ARG targets, *aph(3’)-IIIa*, *ermB*, *qnrS*, *sul1*, and *tetM* were detected in all nucleic acid samples, whereas *bla_KPC_* and *vanA* were not detected in any of the samples. *Bla_CTX_M-1_* was detected in 84.1% of the samples (*n* = 37) collected from all aircraft, except those from the UK (*n* = 4) and Germany (*n* = 3). *Bla_NDM-1_* was mainly detected in aircraft wastewater samples originating from India (*n* = 8), UK (*n* = 5), Germany (*n* = 2), and single flights departing from Japan and South Africa. Concentrations in order of the highest to lowest, median (IQR) in log_10_ - transformed GC/L were 9.99 (9.02 to 12.2) for *aph(3’)-IIIa*, 0 (0 to 8.57) for *bla_NDM-1_*, 8.10 (0 to 10.0) for *bla_CTX_M-1_*, 11.6 (10.1 to 12.6) for *ermB*, 8.32 (6.70 to 10.4) for *qnrS*, 11.7 (10.2 to 12.8) for *sul1* and 11.2 (8.54 to 12.3) for *tetM*. To account for the potential bias in ARG profiles introduced by defecation patterns among flights, the 16S rRNA gene, ENT 23S rRNA gene, and three human fecal markers (HF183, pBI143, and *Carjivirus*) were used as the independent normalization factors to calculate the relative abundances of nine ARGs, which were then subjected to PCA. The explained variance ratios by the first two principal components (PC1 and PC2) showed distinct differences depending on the normalization factor used (Table S6).

**Fig 4:**
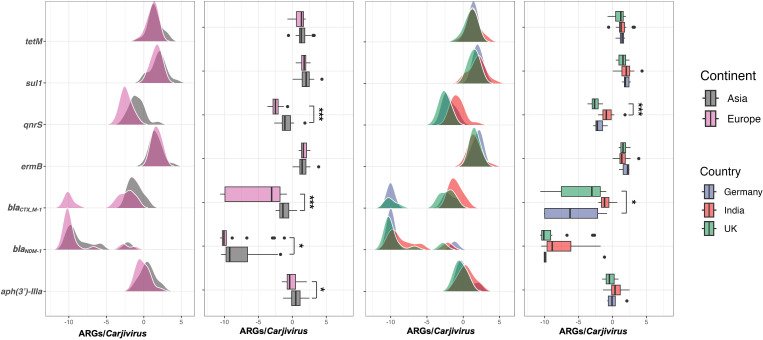
Normalized ARGs across flights differentiated by continents and countries (* indicates *P* < 0.05).

The highest explained variance for the first two components was observed for using *Carjivirus* as the normalization factor (PC1 and PC2 explained 72.9% of the total variance), followed by pBI143 (69.3%), ENT 23 rRNA gene (67.9%), HF183 (63.4%) and 16S rRNA gene (51.8%). *Carjivirus* was used as a normalization factor to enable the comparison of ARG prevalence across different flights. Significant differences were observed for relative abundances of *bla_CTX_M-1_* (*P* < 0.001) and *qnrS* (*P* < 0.001) in flights departing from India, UK, and Germany (Fig. 4). Significantly higher abundances of *bla_CTX_M-1_* in aircraft wastewater samples were observed in flights from India compared to those from the UK and Germany (*P* < 0.05). Meanwhile, significantly higher abundances of *qnrS* were found in flights from India compared to those from the UK (*P* < 0.001). When samples were aggregated based on continents of departure, wastewater in flights from Asia exhibited significantly higher abundances of *aph(3′)-IIIa* (*P* < 0.05), *bla_NDM-1_* (*P* < 0.05), *bla_CTX_M-1_* (*P* < 0.001), and *qnrS* (*P* < 0.001) than those from Europe.

### Correlations among human fecal/urine markers, pathogens, and ARGs from aggregated aircraft wastewater samples

Statistical analysis revealed an overall significant correlation pattern between the concentrations of targeted ARGs with at least one of the human fecal/urine markers screened in this study (*P* < 0.05) except for *bla_NDM-1_* (Fig. S3). While the correlation among the concentrations of ARGs and five ESKAPE pathogens was only significant between *E. faecium* and ARGs *aph(3′)-IIIa* (*r* = 0.31), *ermB* (*r* = 0.37) and *sul1* (*r* = 0.34) (*P* < 0.05), and *S. aureus* with *aph(3’)-IIIa* (*P* < 0.05, *r* = 0.34). In addition, significant correlations were found among ARG concentrations for *bla_NDM-1_*, *bla_CTX_M-1_*, and *qnrS* (*P* < 0.05) and *aph(3’)-IIIa*, *qnrS*, *sul1,* and *tetM* (*P* < 0.05).

## DISCUSSION

International flights serve as a converging vector for the global transmission of AMR and other pathogens, resulting in the importation of AMR via humans originating from diverse geographic regions to the local environment (14, 53). The potential of aircraft-based wastewater monitoring to track the global transmission of AMR has been highlighted to enhance global AMR surveillance and management (54). However, uncertainties remain regarding the efficiency and representativeness of the surveillance data in accurately reflecting the AMR burden among flight passengers (7). In this proof-of-concept study, wastewater samples analyzed were collected directly from aircraft lavatories from individual flights. Such an approach could establish a more targeted surveillance framework, facilitating high-resolution mapping of AMR spread across international borders compared to sampling from vacuum trucks or airport sewer outlets. Wastewaters from post-flight composite sources are likely to be diluted compared to lavatory wastewater samples taken directly from individual aircraft and may not represent a specific population group arriving from a particular destination (19).

By screening lavatory wastewater samples from 44 repatriation flights to Australia, we were able to monitor and quantify FIB, several human fecal/urine markers, putative ESKAPE pathogens, and clinically relevant ARGs originating from different countries/continents of departure using qPCR. The prevalence of two FIBs and four fecal/urine marker genes confirmed the use of lavatories by the passengers and crew onboard, which varied between samples from different flight origins. The high abundance of pBI143 in flights departing from different countries also suggested the widespread presence of this plasmid in human guts (55). Significantly lower concentrations of fecal markers (ENT 23S rRNA, HF183, pBI143 and *Carjivirus*) were detected in flights departing from Asia (*n* = 20), including 18 flights from India, compared to those departing from Europe (*n* = 21), which comprised 14 flights from the UK and Germany. Noting the shorter flight durations from Asia (2 h & 13 min to 9 h & 38 min) compared to those from Europe (15 h & 20 min to 15 h & 49 min) (Supplemental Table ST1), the results suggested relatively higher fecal markers on longer-haul flights, which aligned with the likelihood of defecation onboard as indicated by a survey (56). However, various factors, including the timing of food service, passenger demographics, and other flight conditions such as the availability of lavatories and passenger comfort, could influence toilet usage. For HPyV, which is primarily excreted in the urine and/or feces, its significant correlation with the number of passengers, along with the lack of significant concentration differences between flights from Asia and Europe, might be attributed to the generally higher likelihood of urination compared to defecation among passengers during flights (57). The high frequency of urination was also reflected in the comparatively high abundance of common uropathogens, such as *K. pneumoniae* and *E. faecium*, among the pathogens screened in aircraft lavatory wastewater samples (21). It is important to note that while these organisms are frequently associated with infections, they can also be part of the healthy microbiome and urethra in individuals, and AMR genes may be present in both pathogenic and non-pathogenic strains in the gut microbiome. Thus, the presence of these organisms in wastewater samples does not necessarily imply pathogenicity in all cases, but rather reflects the broader diversity of bacteria in the human microbiome, including those harboring AMR genes.

The detection of ARGs, including several clinically important *aph(3’)-IIIa*, *bla_NDM-1_*, *bla_CTX_M-1_*, *ermB,* and *qnrS*, in aircraft wastewater samples underscores the potential dissemination of AMR across international borders. To enable comparison across flights departing from different countries or continents, individual ARG numbers were normalized using *Carjivirus* GC to reduce potential bias from variations in passenger defecation frequency. Specifically, significantly higher abundances of *bla_CTX_M-1_* and *qnrS* were found in aircraft wastewater samples from India compared to those from the UK and Germany, suggesting a potential higher importation of these ARGs among passengers travelling to Australia. *Bla_CTX_M-1_* and *qnrS* have been found to coexist on the same plasmid (58). The co-occurrence of these two ARGs in aircraft wastewater samples was further supported by correlation analysis. Previous studies on the gut microbiome of healthy travelers also showed that travel to the Indian subcontinent was associated with the highest acquisition rates of *bla_CTX_M-1_* and *qnrS* (23, 59). Additionally, the acquisition rates of extended-spectrum β-lactamase-producing *Enterobacteriaceae* (ESBL-E), which encode the CTX-M, SHV, and TEM enzymes, were found to be the highest in India among other Asian and European countries in a prospective cohort study (22). Among the ESBL genes detected in fecal samples by travellers, bla*_CTX_M-1_* was one of the most frequently acquired genes (22, 59). When aircraft wastewater samples were aggregated based on the continents of departure, flights from Asia exhibited an overall higher abundance of *aph(3’)-IIIa*, *bla_NDM-1_*, *bla_CTX_M-1_*, and *qnrS* than those from Europe. This finding aligns with previous studies suggesting higher AMR levels in South Asia than in Europe, as indicated by the resistome from lavatory waste on long-distance flights (25). This difference is likely driven by the widespread dissemination of specific ARGs, dietary changes, potential food contamination, water sanitation, and hygiene practices in the departure countries (22). In addition, the comparatively higher levels of AMR genes were also reflected in urban sewage samples from Asia, where distinct geographic signatures in the resistome were identified both at the city and world region level among metagenomic sequencing data pooled from more than 100 countries (60). In the context of global traveling and migration, certain geographies may be more prone to transmission events of ARGs due to factors such as inadequate wastewater treatment, high population density, and increased antibiotic use, and thus need additional attention (60, 61). Therefore, aircraft-based wastewater surveillance could serve as a complementary tool for monitoring global transmission of ARGs and mapping AMR hotspots across the world. Such surveillance efforts are anticipated to provide critical data to inform local health authorities and international health agencies like the World Health Organization (WHO).

Previous studies comparing ARG profiles between airplane wastewater and local urban wastewater suggest that the potential risk (indicated by analysis of aircraft wastewater) lies in the dissemination of imported ARGs, which are rarely found in local environmental systems (19). The *bla_NDM-1_* gene, which was detected in 17 aircraft lavatory samples, could not be detected in urban Australian wastewater samples collected in 2020 and 2021 (data not shown). This finding suggests the potential introduction of this ARG to the local wastewater system through international travel.

This gene could subsequently be introduced into the local aquatic environment, given that ARGs are only partially removed during wastewater treatment processes before discharge (62). It is also crucial to consider how ARGs can persist and spread within the microbiomes of travelers. Upon arrival in a new country, these genes may continue to propagate in individuals' gut microbiomes, potentially leading to the establishment of resistant strains. The introduction of these genes through international travel can contribute to the long-term persistence and spread of AMR in local populations, highlighting the need for monitoring both the initial introduction and the ongoing transmission of AMR. This highlights the true value of WBE as a surveillance tool to monitor and mitigate the risks associated with AMR importation, emphasizing the need for robust strategies to address the global movement of resistant genes and their potential integration into domestic environments. Ideally, a comparison between the abundance of ARGs in lavatory wastewater from inbound flights and their counterparts in influent wastewater from Australian WWTPs would provide a domestic baseline to assess whether inbound flights may contribute to new or elevated burdens of AMR. However, such baseline data were not available for the WWTPs where the aircraft landed.

A notable challenge for implementing aircraft wastewater surveillance is the use of disinfectants, which can potentially degrade nucleic acids and hinder the detection of microbial markers (7). While limited data exist on SARS-CoV-2 degradation in aircraft lavatory wastewater (63), no information is available on the degradation of bacteria and ARGs. Understanding the loss of nucleic acid targets is crucial to determining whether such a surveillance system can be effective in detecting clinically significant targets. Our findings suggest that the concentrations of several representative targets, such as *Carjivirus*, ENT 23S rRNA, and *tetM*, remained stable over a 24 h incubation with added disinfectants, even in the presence of the highest concentrations of disinfectants. This indicates that nucleic acids are resilient enough to persist in aircraft wastewater over the maximum duration of a flight. The minimal degradation observed in our study suggests that aircraft wastewater samples can be reliably used for AMR surveillance, even when collected several hours after passenger disembarkation.

This study underscores the potential of aircraft lavatory wastewater surveillance for assessing the prevalence of fecal indicator bacteria (FIB), human fecal/urine markers, bacterial pathogens, and antibiotic resistance genes (ARGs) on individual flights. By revealing the complex interplay between fecal/urine contamination, pathogen presence, and ARG dissemination, the findings highlight the feasibility of leveraging this approach to monitor public health threats.

It is important to note that the samples analyzed in this study were collected during the COVID-19 pandemic, when global travel was significantly restricted, and the flights sampled were designated repatriation flights, primarily carrying Australian citizens and residents. These unique circumstances may limit the representativeness of the findings for typical air travel conditions. The restricted passenger demographics and altered travel patterns during this period could have resulted in less cross-border exchange of ARG profiles. Consequently, the geographic differences observed in this study might be attributed to these pandemic-specific factors. Further studies under normal travel conditions will be necessary to validate these findings and evaluate the global applicability of aircraft wastewater surveillance for ARG monitoring. The presence of ARGs or pathogen genes does not necessarily indicate viable or infectious organisms, since qPCR was used. Low Cq values in aircraft wastewater samples for certain targets suggested the presence of trace amounts of genetic material. However, these traces could stem from previous flights, leading to potential carryover contamination.

While wastewater surveillance for SARS-CoV-2 on long-haul flights has demonstrated high predictive value for detecting COVID-19 infections and guiding quarantine measures, applying similar methodologies to AMR surveillance requires careful adaptation. Unlike SARS-CoV-2, which spreads rapidly and causes symptoms to develop quickly, AMR is a "silent pandemic" with less immediate public health implications, necessitating distinct strategies and follow-up actions. AMR surveillance at borders must consider long-term consequences, including the potential for colonization without symptoms and the need for coordinated responses across health sectors.

Aircraft passengers may represent a subpopulation reflective of the broader regions they originate from. Developing a global aircraft-based wastewater surveillance network could provide early warnings of emerging microbial threats across geographical boundaries, including the spread of (novel) ARGs from specific hotspots. Future studies should prioritize integrating advanced methodologies, such as metagenomic sequencing, to identify novel ARGs and pathogens. This approach would provide a comprehensive understanding of AMR transmission mechanisms, enhance global disease surveillance capabilities, and inform tailored interventions.

In conclusion, this study demonstrates the potential of aircraft lavatory wastewater as a valuable tool for monitoring global AMR circulation. By capturing high-resolution data on FIB, human fecal/urine markers, pathogens, and ARGs from specific flight populations, we highlight its feasibility for tracking the cross-border spread of AMR. Our findings underscore geographic differences in ARG prevalence, aligning with known regional disparities in AMR burden. Despite challenges like disinfectant use, the stability of nucleic acids reinforces the reliability of this approach. Aircraft wastewater surveillance could complement global health efforts, offering early warnings of emerging AMR threats and informing targeted interventions. Future studies under normal travel conditions will be critical to refining this method and enhancing global AMR surveillance. Furthermore, integrating advanced techniques such as sequencing will be essential to detect a wide range of microbial taxa and characterize novel ARGs with high sensitivity, thus improving the accuracy and depth of AMR monitoring. By leveraging these tools, we can better identify emerging pathogens and resistance genes, enabling more effective and timely interventions in AMR management.

## AFTER EPUB

*[This article was published on 28 May 2025 with errors in the Acknowledgments. The Acknowledgments were corrected in the current version, posted on 5 June 2025.]*

## SUPPLEMENTAL MATERIAL

Supplemental material spectrum.00569-25-s0001.docx Tables S1 to S6; Fig. S1 to S3.

ASM does not own the copyrights to Supplemental Material that may be linked to, or accessed through, an article. The authors have granted ASM a non-exclusive, world-wide license to publish the Supplemental Material files. Please contact the corresponding author directly for reuse.
