# Supplementary material for: Aircraft lavatory wastewater surveillance for movement of antimicrobial resistance genes: a proof-of-concept study
Source: Microbiol Spectr. 2025 May 28;13(7):e00569-25. doi: 10.1128/spectrum.00569-25 (PMC12211000; doi:10.1128/spectrum.00569-25)
Supplement: Supplemental material — Tables S1 to S6; Fig. S1 to S3. [file spectrum.00569-25-s0001.docx]

**Aircraft lavatory wastewater surveillance for movement of antimicrobial resistance genes: a proof-of-concept study**

Yawen Liu^1,2^, Wendy J.M. Smith^2^, Metasebia Gebrewold^2^, Nicholas J. Ashbolt^3^, Ishi Keenum^4^, Stuart L. Simpson^2^, Xinhong Wang^1^, Warish Ahmed^2^

1. State Key Laboratory of Marine Environmental Science, College of the Environment & Ecology, Xiamen University, Xiamen 361102, China.

2. CSIRO Environment, Ecosciences Precinct, 41 Boggo Road, Dutton Park, QLD 4102, Australia.

3. Future Industries Institute, University of South Australia, and Cooperative Research Centre for Solving Antimicrobial Resistance in Agribusiness, Foods and Environments, Pooraka, SA 5095, Australia

4. Department of Civil, Environmental, and Geospatial Engineering. Michigan Technological University, Houghton, MI 49931, USA

**Corresponding author.** Warish Ahmed. Mailing address: Ecosciences Precinct, 41 Boggo Road, Dutton Park 4102, Queensland, Australia. Tel.: +617 3833 5582; E-mail address: [Warish.Ahmed@csiro.au](mailto:Warish.Ahmed@csiro.au)

**Supplementary Materials**

**Supplementary Table ST1**

**General information of aircraft wastewater samples collected from repatriation flights in Australia**

| Sample ID | Departure country | Sampling date (day/mo/year) | Passengers | Flight duration |
| --- | --- | --- | --- | --- |
| UK_2 | UK | 8/NOV/2020 | 175 | 15 h & 49 min |
| UK_8 | UK | 12/NOV/2020 | 175 | 15 h & 49 min |
| India_12 | India | 24/ NOV/2020 | 175 | 9 h & 38 min |
| India_13 | India | 28/ NOV /2020 | 182 | 9h & 38 min |
| Germany_3 | Germany | 13/DEC/2020 | 170 | 15 h & 24 min |
| France | France | 17/ DEC/2020 | 150 | 15h & 20min |
| Germany_4 | Germany | 3/JAN/2021 | 175 | 15 h & 24 min |
| India_17 | India | 8/JAN/2021 | 150 | 9 h & 38 min |
| India_14 | India | 19/JAN/2021 | 163 | 9 h & 38 min |
| India_15 | India | 20/MAR/2021 | 160 | 9 h & 38 min |
| UK_11 | UK | 31/JAN/2021 | 138 | 15h & 49min |
| UK_12 | UK | 17/FEB/2021 | 190 | 15 h & 49 min |
| UK_13 | UK | 19/FEB/2021 | 193 | 15 h & 49 min |
| Germany_5 | Germany | 21/FEB/2021 | 195 | 15 h & 24 min |
| UK_14 | UK | 23/FEB/2021 | 195 | 15 h & 49 min |
| Germany_6 | Germany | 31/MAR/2021 | 120 | 15 h & 24 min |
| India_18 | India | 15/APR/2021 | 165 | 9 h & 38 min |
| India_16 | India | 17/APR/2021 | 169 | 9 h & 38 min |
| UK_1 | UK | 17/APR/2021 | 150 | 15 h & 49 min |
| India_1 | India | 25/MAY/2021 | 140 | 9 h & 38 min |
| India_2 | India | 25/MAY/2021 | 141 | 9 h & 38 min |
| Turkey | Turkey | 24/MAY/2021 | 120 | 15 h & 12 min |
| India_3 | India | 29/MAY/2021 | 150 | 9 h & 38 min |
| UK_3 | UK | 25/MAY/2021 | 150 | 15 h & 49 min |
| India_4 | India | 4/JUN/2021 | 160 | 9 h & 38 min |
| India_5 | India | 6/JUL/2021 | 170 | 9 h & 38 min |
| India_6 | India | 11/JUL/2021 | 183 | 9 h & 38 min |
| Germany_1 | Germany | 13/JUL/2021 | 150 | 15 h & 24 min |
| UK_4 | UK | 22/JUL/2021 | 175 | 15 h & 49 min |
| UK_5 | UK | 24/JUL/2021 | 150 | 15 h & 49 min |
| India_7 | India | 1/AUG/2021 | 181 | 9 h & 38 min |
| UK_6 | UK | 2/AUG/2021 | 181 | 15 h & 49 min |
| Japan | Japan | 3/AUG/2021 | 120 | 6h & 39min |
| UK_7 | UK | 4/AUG/2021 | 180 | 15 h & 49 min |
| India_8 | India | 10/AUG/2021 | 175 | 9 h & 38 min |
| India_9 | India | 11/AUG/2021 | 175 | 9 h & 38 min |
| UK_9 | UK | 12/AUG/2021 | 165 | 15 h & 49 min |
| Germany_2 | Germany | 13/AUG/2021 | 170 | 15 h & 24 min |
| Indonesia | Indonesia | 18/AUG/2021 | 180 | 2 h & 13 min |
| South Africa | South Africa | 19/AUG/2021 | 166 | 11 h & 1 min |
| India_10 | India | 21/AUG/2021 | 180 | 9 h & 38 min |
| UK_10 | UK | 22/AUG/2021 | 170 | 15 h & 49 min |
| India_11 | India | 7/SEP/2021 | 150 | 9 h & 38 min |
| Dubai | UAE | 7/SEP/2021 | 94 | 13 h & 25 min |

UK: United Kingdom; UAE: United Arab Emirates.

**Supplementary Table ST2**

**qPCR primer and probe concentrations and cycling parameters used in this study.**

| Targets [references] | Primers and probes (5’ - 3’) | Primer and probe concentrations (nM) | Cycling parameters |
| --- | --- | --- | --- |
| Bacteria 16S RNA [1] | F: ATG GYT GTC GTC AGC T | 300 | 95°C for 10 min, 40 cycles of 15 s at 95 °C,60 s at 52 °C |
|  | R: ACG GGC GGT GTG TAC | 300 |  |
|  | P: FAM-CAA CGA GCG CAA CCC-TAMRA | 300 |  |
| *E. coli* 23S rRNA gene [2] | F: GGT AGA GCA CTG TTT TGG CA | 800 | 10 min at 95 °C, 40 cycles of 15 s at 95 °C, 60 s at 60 °C |
|  | R: TGT CTC CCG TGA TAA CTT TCTC | 800 |  |
|  | P: FAM-TCATCCCGACTTACCAACCCG-TAMRA | 80 |  |
| *Enterococcus* spp. 23S rRNA gene [3] | F: AGA AAT TCC AAA CGA ACT TTG | 500 | 10 min at 95 °C, 40 cycles of 15 s at 95 °C, 2 min at 60 °C |
|  | R: CAG TGC TCT ACC TCC ATC ATT | 500 |  |
|  | P: FAM-TGG TTC TCT CCG AAA TAG CTT TAG GGC TA-TAMRA | 400 |  |
| *Bacteroides* HF183 [4] | F: GGT TTC CGC AGC TGG G | 1000 | 95°C for 10 min, 45 cycles of 95°C for 15 s, 60°C for 60 s |
|  | R: CCG AGC CGT CCT GGT CTA | 1000 |  |
|  | P: FAM-AGT CGC AGG CGG CCA CCG T-TAMRA | 100 |  |
| pBI143 [5] | F: ACG ACA ATT ATC TGG CTT TG | 400 | 95°C for 10 min, 45 cycles of 15 s at 95 ℃, 60 s at 56 ℃ |
|  | R: AGC GAA CCT TGC CTT TCA | 400 |  |
|  | P: FAM-CAT TGA GTG AAG CTG CCG TTT GGT-BHQ1 | 400 |  |
| *Carjivirus* (formerly known as CrAssphage) [6] | F: CAG AAG TAC AAA CTC CTA AAA AAC GTA GAG | 1000 | 95°C for 10 min, 45 cycles of 95°C for 15 s, 60°C for 60 s. |
|  | R: AT GAC CAA TAA ACA AGC CAT TAG C | 1000 |  |
|  | P: FAM-AAT AAC GAT TTA CGT GAT GTA AC-TAMRA | 100 |  |
| Human polyomavirus (HPyV) [7] | F: AGT CTT TAG GGT CTT CTA CCT TT | 240 | 95°C for 10 min, 40 cycles of 30 s at 95°C, 20 s at 55°C and 60 s at 60°C |
|  | R: GGT GCC AAC CTA TGG AAC AG | 240 |  |
|  | P: FAM-TCA TCAC TGG CAA ACA T-MGBNFQ | 160 |  |
| *E. faecium* (ddl gene) [8] | F: TAA ACT TCT TCC GGC ACT TCG | 400 | 10 min at 95 ℃, 45 cycles of 15 s at 95℃,60 s at 60 ℃ |
|  | R: TGC TTT AGC AAC AGC CTA TCA G | 400 |  |
|  | P: FAM-CTC GAG CAA TCG TTG AAC AAG GAA TTG-BHQ-2 | 200 |  |
| S. aureus (nuc gene) [9] | F: GCG ATT GAT GGT GAT ACG GTT | 400 | 10 min at 95 ℃, 45 cycles of 15 s at 95℃,60 s at 60 ℃ |
|  | R: AGC CAA GCC TTG ACG AAC TAA AGC | 400 |  |
|  | P: FAM-GGT GTA GAG AAA TAT GGT CCT GAA GCA AGT GCA-BHQ1 | 100 |  |
| K. pneumoniae (khe gene) [10] | F: CGA TGC TAC TTA TCC CGA CA | 300 | 10 min at 95 ℃, 45 cycles of 15 s at 95℃,60 s at 60 ℃ |
|  | R: AGC CGG TTG AGA CGT AAA C | 300 |  |
|  | P: FAM-CCG ATT GAA AAA CGC TCC GGG C -BHQ1 | 200 |  |
| A. baumannii (bap gene) [11] | F: CGC TGC AGC ATC AAA TCA TG | 900 | 10 min at 95 ℃, 45 cycles of 15 s at 95℃,60 s at 60 ℃ |
|  | R: TGG GTC AAC CGA GAA AGT TAC G | 900 |  |
|  | P: FAM-AGC ACC TGC TGA CAC CAC TCC ACC A-TAMRA | 250 |  |
| P. aeruginosa (gyrb gene) [12] | F: CCT GAC CAT CCG TCG CCA CAA C | 400 | 10 min at 95 ℃, 45 cycles of 15 s at 95℃,60 s at 60 ℃ |
|  | R: CGC AGC AGG ATG CCG ACG CC | 400 |  |
|  | P: FAM-CCG TGG TGG TAG ACC TGT TCC CAG ACC-BHQ1 | 200 |  |
| *Mycobacterium* spp. (23S rRNA) [13] | F: GGG GTG TGG TGT TTG AG | 400 | 10 min at 95 ℃, 45 cycles of 30 s at 95℃,40 s at 55 ℃, 30 s at 72 ℃ |
|  | R: CTC CCA CGT CCT TCA TC | 400 |  |
|  | P: FAM-TGG ATA GTG GTT GCG AGC ATC-BHQ1 | 200 |  |
| *Salmonella* spp. [14] | F: CTC ACC AGG AGA TTA CAA CAT GG | 400 | 10 min at 95 ℃, 45 cycles of 15 s at 95℃,30 s at 65 ℃ |
|  | R: GA TGG TCA CTT TTG GTC TGA GCT | 400 |  |
|  | P: FAM-CTA ATT TAA CCC GTC GTC AGT GGC T-BHQ | 200 |  |
| aph(3’)-IIIa [15] | F: ACA TAT CGG ATT GTC CCT ATA CGA A | 1000 | 10 min at 95 ℃, 45 cycles of 15 s at 95℃,60 s at 56 ℃ |
|  | R: TCG GCC AGA TCG TTA TTC AGT A | 1000 |  |
|  | P: FAM-TAG CTT AGA CAG CCG CTT A-BHQ1 | 500 |  |
| blaNDM-1 [16] | F: ATT AGC CGC TGC ATT GAT | 500 | 95°C for 10 min, 45 cycles of 15 s at 95 ℃, 60 s at 60 ℃ |
|  | R: CAT GTC GAG ATA GGA AGT G | 500 |  |
|  | P: FAM-AGC AAA TGG AAA CTG GCG ACC AAC -TAMRA | 100 |  |
| blaCTX_M-1 [17] | F: ACC AAC GAT ATC GCG GTG AT | 1000 | 95°C for 10 min, 45 cycles of 15 s at 95 ℃, 60 s at 60 ℃ |
|  | R: ACA TCG CGA CGG CTT TCT | 1000 |  |
|  | P: FAM- TCG TGC GCC GCT G -TAMRA | 200 |  |
| blaKPC [18] | F: GAT ACC ACG TTC CGT CTG G | 900 | 10 min at 95 ℃, 45 cycles of 15 s at 95℃,60 s at 60 ℃ |
|  | R: GCA GGT TCC GGT TTT GTC TC | 900 |  |
|  | P: FAM-CGG CAG CAG TTT GTT GAT TGG CTA-BHQ1 | 500 |  |
| ermB [19] | F: GAT ACC GTT TAC GAA ATT GG | 300 | 10 min at 95℃, 45 cycles of 15 s at 95℃,30 s at 60 ℃, 30 s at 72℃ |
|  | R: GAA TCG AGA CTT GAG TGT GC | 300 |  |
|  | P: FAM-AGC CAG TTT CGT CGT TAA ATG CCC-BHQ1 | 100 |  |
| qnrS [20] | F: CGA CGT GCT AAC TTG CGT GA | 300 | 10 min at 95 ℃, 45 cycles of 15 s at 95℃,60 s at 60 ℃ |
|  | R: GGC ATT GTT GGA AAC TTG CA | 300 |  |
|  | P: FAM-AGT TCA TTG AAC AGG GTG A-BHQ1 | 200 |  |
| tetM [21] | F: GTG GAC AAA GGT ACA ACG AG | 500 | 10 min at 95 ℃, 45 cycles of 15 s at 95℃,60 s at 55 ℃ |
|  | R: CGG TAA AGT TCG TCA CAC AC | 500 |  |
|  | P: ACG AAG GTG AAC ATC ATA GAC ACG CC | 100 |  |
| sul1 [22] | F: CCG TTG GCC TTC CTG TAA AG | 800 | 10 min at 95 ℃, 45 cycles of 15 s at 95℃,60 s at 60 ℃ |
|  | R: TTG CCG ATC GCG TGA AGT | 800 |  |
|  | P: FAM-CAG CGA GCC TTG CGG CGG-TAAMRA | 100 |  |
| vanA [23] | F: GTA GGC TGC GAT ATT CAA AGC | 1000 | 10 min at 95 °C, 40 cycles of 30 s at 95 °C,60 s at 60 °C |
|  | R: CGA TTC AAT TGC GTA GTC CAA | 1000 |  |
|  | P: FAM- TAA AGA TGA TAG GCC GGT GGC AGC-3BHQ1 | 100 |  |

**Supplementary Table ST3**

**qPCR performance characteristics**

| Assays | Efficiency (E) (%) | Linearity (*R*^2^) | Slope | Y-intercept |
| --- | --- | --- | --- | --- |
| Bacteria 16S rRNA | 104.1 | 0.95 | -3.23 | -39.12 |
| *E. coli* 23S rRNA | 98.4 | 0.99 | -3.33 | 38.27 |
| *Enterococcus* spp. 23S rRNA gene | 99.6 | 0.98 | -3.32 | 38.31 |
| *Bacteroides* HF183 | 93.5 | 0.99 | -3.49 | 38.37 |
| pBI143 | 99.7 | 0.99 | -3.33 | 38.55 |
| *Carjivirus* | 100.4 | 0.99 | -3.31 | 40.23 |
| Human polyomavirus (HPyV) | 102.6 | 0.98 | -3.26 | 39.40 |
| *E. faecium* | 96.9 | 1 | -3.40 | 38.04 |
| *S. aureus* | 91.3 | 0.99 | -3.55 | 39.66 |
| K. pneumoniae (khe gene) | 100.9 | 1 | 3.30 | 36.91 |
| A. baumannii (bap gene) | 98.9 | 0.99 | -3.35 | 36.73 |
| P.aeruginosa (gyrb gene) | 98.7 | 0.99 | -3.35 | 36.25 |
| *Mycobacterium* spp. | 96.4 | 0.98 | -3.49 | 38.37 |
| *Salmonella* spp. | 96.9 | 0.99 | -3.45 | 33.73 |
| aph(3’)-IIIa | 90.2 | 0.99 | -3.59 | 40.22 |
| *bla_NDM-1_* | 90.1 | 1.00 | -3.58 | 40.5 |
| *bla_CTX_M-1_* | 91.3 | 0.96 | -3.55 | 45.9 |
| blaKPC | 96.7 | 0.99 | -3.46 | 39.87 |
| *ermB* | 97.7 | 0.99 | -3.38 | 38.8 |
| *qnrS* | 110 | 0.94 | -3.10 | 41.8 |
| *tetM* | 96.6 | 0.99 | -3.47 | 39.40 |
| *sul1* | 98.5 | 0.99 | -3.37 | 42.10 |
| *vanA* | 91.0 | 0.99 | -3.56 | 38.44 |

**Supplementary Table ST4**

**Concentrations of bacteria 16S rRNA gene and six human fecal/urine markers in aircraft wastewater samples.**

| Aircraft wastewater samples | Concentrations (Log_10_ GC/L) | | | | | | |
| --- | --- | --- | --- | --- | --- | --- | --- |
|  | Bacteria 16S rRNA | *E. coli* 23S rRNA | *Enterococcus* spp. 23S rRNA | *Bacteroides* HF183 | pBI143 | *Carjivirus* | Human polyomavirus (HPyV) |
| France | 13.21 | 7.7 | 11.67 | 9.31 | 10.91 | 10.32 | 7.22 |
| Germany_1 | 12.85 | 9.04 | 11.36 | 8.58 | 10.3 | 9.71 | 7.67 |
| Germany_2 | 12.85 | 8.78 | 11.3 | 8.59 | 9.24 | 10.03 | 7.89 |
| Germany_3 | 12.7 | 8.13 | 9.59 | 8.18 | 8.89 | 10.09 | 7.82 |
| Germany_4 | 12.7 | 7.62 | 9.11 | 8.16 | 9.19 | 9.74 | 7.36 |
| Germany_5 | 13.37 | 7.94 | 11.34 | 8.48 | 9.99 | 10.1 | 7.44 |
| Germany_6 | 12.87 | 8.09 | 10.97 | 8.67 | 9.96 | 9.94 | 7.77 |
| UK_1 | 12.6 | 9.47 | 10.67 | 8.11 | 9.86 | 10.68 | 6.06 |
| UK_2 | 11.9 | 7.56 | 10.03 | 8.58 | 9.63 | 10.15 | 7.37 |
| UK_3 | 12.42 | 8.64 | 10.53 | 8.13 | 10 | 8.87 | 7.73 |
| UK_4 | 13.01 | 7.86 | 11.1 | 8.55 | 9.51 | 9.97 | 7.52 |
| UK_5 | 12.96 | 7.75 | 11.21 | 8.69 | 10.09 | 10.26 | 7.74 |
| UK_6 | 12.98 | 7.81 | 11.34 | 9.24 | 10.58 | 9.83 | 7.34 |
| UK_7 | 12.72 | 7.66 | 10.17 | 8.66 | 9.91 | 9.87 | 7.88 |
| UK_8 | 12.79 | 8.01 | 10.75 | 9.05 | 10.29 | 10.62 | 6.78 |
| UK_9 | 12.97 | 8.12 | 11.18 | 8.79 | 10.36 | 10.45 | 7.9 |
| UK_10 | 12.44 | 8.36 | 9.13 | 7.95 | 9.15 | 10.42 | 7.42 |
| UK_11 | 12.49 | 6.72 | 9.83 | 8.26 | 9.52 | 9.97 | 7.02 |
| UK_12 | 12.99 | 7.12 | 10.24 | 8.91 | 10.41 | 10.12 | 7.18 |
| UK_13 | 12.53 | 7.42 | 11.22 | 8.2 | 9.59 | 10.64 | 5.52 |
| UK_14 | 13.04 | 8.36 | 10.76 | 9.28 | 10.39 | 10.28 | 7.24 |
| Dubai | 12.49 | 7.85 | 10.23 | 7.29 | 9.4 | 10.49 | 4.06 |
| Turkey | 12.76 | 8.68 | 11.17 | 8.96 | 10.67 | 10.51 | 6.32 |
| India_1 | 12 | 8.32 | 9.19 | 8.31 | 9.62 | 9.5 | 5.98 |
| India_2 | 12.38 | 8.6 | 9.27 | 8.36 | 9.26 | 7.54 | 7.43 |
| India_3 | 12.62 | 9.4 | 10.24 | 8.59 | 10.78 | 9.68 | 6.91 |
| India_4 | 12.59 | 8.43 | 10.11 | 8.16 | 9.95 | 9.42 | 7.46 |
| India_5 | 12.86 | 9.87 | 9.76 | 8.74 | 10.42 | 10.96 | 7.76 |
| India_6 | 12.15 | 8.29 | 9.44 | 7.53 | 8.8 | 10.31 | 6.58 |
| India_7 | 12.35 | 8.5 | 9.71 | 7.38 | 8.73 | 10.27 | 7.65 |
| India_8 | 12.46 | 8.76 | 9.88 | 7.89 | 8.87 | 10.51 | 7.68 |
| India_9 | 12.91 | 8.66 | 11.24 | 9.02 | 9.86 | 10.09 | 8.03 |
| India_10 | 12.91 | 8.48 | 10.52 | 8.35 | 9.17 | 9.51 | 7.03 |
| India_11 | 12.62 | 8.95 | 9.3 | 8.37 | 9.3 | 10.39 | 7.06 |
| India_12 | 11.85 | 7.92 | 8.76 | 6.8 | 7.98 | 8.59 | 6.57 |
| India_13 | 12.55 | 8.2 | 10.07 | 7.57 | 9.21 | 9.19 | 8.06 |
| India_14 | 12.23 | 7.89 | 9.79 | 7.76 | 9.87 | 9.29 | 6.26 |
| India_15 | 12.92 | 8.31 | 10.1 | 8.08 | 9.42 | 9.55 | 7.79 |
| India_16 | 11.98 | 7.72 | 8.95 | 7.18 | 8.48 | 9.07 | 6.56 |
| India_17 | 12.63 | 9.05 | 10.47 | 7.89 | 9.41 | 10.11 | 7.67 |
| India_18 | 12.88 | 7.63 | 10.77 | 8.5 | 9.83 | 8.04 | 8.02 |
| Indonesia | 12.73 | 7.93 | 10.04 | 7.79 | 9.53 | 10.27 | 7.61 |
| Japan | 13.31 | 9.09 | 9.57 | 9.4 | 12.02 | 9.6 | 7.6 |
| South Africa | 13.02 | 7.73 | 11.01 | 9.89 | 11.35 | 9.25 | 8.34 |

**Supplementary Table ST5**

**Concentrations of five ESKPAE pathogens and nine ARGs in aircraft wastewater samples.**

| Aircraft wastewater samples | Concentrations (Log_10_ GC/L) | | | | | | | | | | | | | |
| --- | --- | --- | --- | --- | --- | --- | --- | --- | --- | --- | --- | --- | --- | --- |
|  | *E. faecium* | *S. aureus* | *K. pneumoniae* (*khe* gene) | *A. baumannii* (*bap* gene) | *P. aeruginosa* (*gyrb* gene) | *Mycobacterium* spp. | *Salmonella* spp. | *aph(3’)-IIIa* | bla_NDM-1_ | bla_CTX_M-1_ | *ermB* | *qnrS* | *sul1* | *tetM* |
| France | 8.37 | 3.35 | 6.28 | 6.46 | 0 | 0 | 0 | 11.01 | 0 | 6.25 | 12.26 | 7.92 | 12.51 | 11.74 |
| Germany_1 | 7.88 | 0 | 7.69 | 0 | 0 | 0 | 0 | 10.18 | 8.53 | 8.91 | 12.09 | 9 | 11.69 | 11.56 |
| Germany_2 | 7.87 | 0 | 8.2 | 0 | 0 | 0 | 0 | 9.63 | 0 | 0 | 12.44 | 7.89 | 11.4 | 11.43 |
| Germany_3 | 6.97 | 0 | 8.96 | 0 | 0 | 0 | 0 | 9.32 | 0 | 0 | 11.04 | 7.6 | 11.9 | 10.54 |
| Germany_4 | 0 | 0 | 7.12 | 7.53 | 0 | 0 | 0 | 9.05 | 0 | 7.16 | 11.15 | 7.42 | 12.33 | 10.81 |
| Germany_5 | 8.14 | 0 | 6.7 | 0 | 0 | 0 | 0 | 12.23 | 0 | 8.05 | 12.59 | 7.23 | 12.8 | 11.54 |
| Germany_6 | 0 | 0 | 6.49 | 3.33 | 0 | 0 | 0 | 10.19 | 0 | 0 | 12.02 | 8.7 | 11.68 | 11.66 |
| UK_1 | 6.46 | 0 | 8 | 3.16 | 0 | 0 | 0 | 9.48 | 0 | 9.72 | 11.61 | 9.12 | 11.59 | 11.11 |
| UK_2 | 7.75 | 0 | 0 | 0 | 0 | 0 | 0 | 9.29 | 0 | 7.09 | 11.76 | 7.52 | 10.65 | 10.5 |
| UK_3 | 7.48 | 0 | 6.86 | 0 | 0 | 0 | 0 | 9.46 | 0 | 0 | 11.53 | 6.77 | 11.29 | 10.83 |
| UK_4 | 9.82 | 0 | 8.06 | 0 | 0 | 0 | 0 | 10.71 | 7.04 | 7.98 | 12.04 | 8.59 | 12.47 | 11.85 |
| UK_5 | 7.72 | 3.26 | 8.52 | 0 | 0 | 0 | 0 | 11.16 | 3.55 | 0 | 12.26 | 7.57 | 12.23 | 11.37 |
| UK_6 | 7.63 | 0 | 7.29 | 0 | 0 | 0 | 0 | 10.17 | 7.17 | 8.02 | 12.35 | 8.07 | 11.7 | 11.81 |
| UK_7 | 7.28 | 0 | 8.2 | 0 | 0 | 0 | 0 | 9.35 | 0 | 8.95 | 11.49 | 7.34 | 11.34 | 11.52 |
| UK_8 | 6.93 | 0 | 0 | 0 | 0 | 0 | 0 | 9.54 | 0 | 7.35 | 11.81 | 7.76 | 12.06 | 9.9 |
| UK_9 | 9.03 | 0 | 8.63 | 0 | 0 | 0 | 0 | 10.42 | 0 | 7.44 | 12.18 | 8.26 | 11.54 | 11.71 |
| UK_10 | 6.86 | 0 | 6.85 | 0 | 0 | 3.09 | 0 | 9.99 | 0 | 8.65 | 11.76 | 7.38 | 11.02 | 11.01 |
| UK_11 | 0 | 0 | 9.15 | 0 | 0 | 0 | 0 | 9.02 | 0 | 6.41 | 11.11 | 6.7 | 11.96 | 11.32 |
| UK_12 | 6.93 | 0 | 0 | 6.76 | 0 | 0 | 0 | 9.64 | 0 | 0 | 11.61 | 7.44 | 11.74 | 11.3 |
| UK_13 | 6.9 | 0 | 7.59 | 7.36 | 0 | 0 | 0 | 9.11 | 0 | 0 | 11.71 | 6.95 | 11.45 | 11.03 |
| UK_14 | 7.14 | 0 | 6.69 | 0 | 0 | 0 | 0 | 9.93 | 0 | 8.49 | 11.67 | 7.19 | 11.94 | 11.69 |
| Dubai | 7.37 | 0 | 7.64 | 0 | 0 | 0 | 0 | 9.42 | 0 | 8.45 | 11.42 | 7.41 | 11.31 | 10.79 |
| Turkey | 7.32 | 0 | 8.63 | 0 | 0 | 0 | 0 | 10.38 | 0 | 8.59 | 12.04 | 7.97 | 12.07 | 11.18 |
| India_1 | 7.1 | 0 | 8.62 | 0 | 0 | 0 | 0 | 9.39 | 0 | 8.06 | 10.43 | 8.18 | 10.87 | 10.41 |
| India_2 | 8.4 | 0 | 8.78 | 0 | 0 | 0 | 0 | 9.88 | 0 | 8.24 | 10.19 | 9.39 | 11.9 | 10.59 |
| India_3 | 6.98 | 0 | 9.07 | 0 | 0 | 0 | 0 | 9.77 | 0 | 9.96 | 11.46 | 8.44 | 11.68 | 11.18 |
| India_4 | 6.44 | 0 | 8.09 | 3.26 | 0 | 0 | 0 | 10.61 | 3.52 | 8.69 | 11.24 | 8.89 | 11.82 | 11.14 |
| India_5 | 7.34 | 0 | 0 | 0 | 6.7 | 0 | 0 | 9.58 | 8.39 | 9.01 | 10.99 | 9.5 | 12.43 | 10.39 |
| India_6 | 0 | 0 | 7.5 | 0 | 0 | 0 | 0 | 9.47 | 8.57 | 8.49 | 10.98 | 8.9 | 10.36 | 10.97 |
| India_7 | 7.36 | 0 | 8.25 | 0 | 0 | 0 | 0 | 9.84 | 3.42 | 8.77 | 11.37 | 8.56 | 10.54 | 10.75 |
| India_8 | 0 | 0 | 0 | 0 | 7.98 | 0 | 0 | 9.55 | 0 | 8.88 | 11.6 | 8.93 | 10.55 | 11.57 |
| India_9 | 7.15 | 0 | 8.21 | 6.87 | 0 | 0 | 0 | 10.31 | 0 | 9.15 | 11.97 | 9.27 | 12.04 | 11.28 |
| India_10 | 7.57 | 0 | 8.86 | 0 | 0 | 0 | 0 | 10.66 | 7.38 | 8.51 | 11.83 | 9.45 | 11.45 | 11.45 |
| India_11 | 7.32 | 0 | 0 | 0 | 0 | 0 | 2.37 | 10.27 | 0 | 8.78 | 11.33 | 8.97 | 11.36 | 11.4 |
| India_12 | 0 | 0 | 7.87 | 0 | 0 | 0 | 0 | 10.38 | 0 | 8.06 | 10.99 | 8.45 | 10.79 | 9.81 |
| India_13 | 6.52 | 0 | 8.14 | 0 | 0 | 0 | 0 | 10.32 | 0 | 7.13 | 11.07 | 9.05 | 12.27 | 10.99 |
| India_14 | 0 | 0 | 7.87 | 0 | 0 | 0 | 0 | 9.74 | 0 | 7.21 | 10.4 | 7.15 | 11.88 | 10.84 |
| India_15 | 7.51 | 0 | 7.97 | 0 | 0 | 0 | 0 | 9.86 | 0 | 8.21 | 11.02 | 8.56 | 12.28 | 11.58 |
| India_16 | 0 | 0 | 8.25 | 7.88 | 0 | 0 | 0 | 9.64 | 3.47 | 8.59 | 10.35 | 8.76 | 11.13 | 10.37 |
| India_17 | 7.9 | 3.4 | 0 | 3.27 | 0 | 0 | 0 | 10.65 | 0 | 9.59 | 11.39 | 9.6 | 12.16 | 11.22 |
| India_18 | 0 | 0 | 7.08 | 6.54 | 2.89 | 0 | 0 | 10.55 | 0 | 8.32 | 11.91 | 8.31 | 11.27 | 11.25 |
| Indonesia | 8.2 | 0 | 8.62 | 0 | 0 | 0 | 0 | 10.9 | 0 | 7.92 | 11.69 | 7.6 | 11.81 | 11.6 |
| Japan | 6.96 | 0 | 6.53 | 0 | 0 | 0 | 0 | 10.73 | 0 | 7.14 | 11.98 | 7.16 | 12.06 | 12.31 |
| South Africa | 8.05 | 0 | 8.26 | 0 | 0 | 0 | 0 | 11.72 | 0 | 7.91 | 11.28 | 8.38 | 12.27 | 11.43 |

**Supplementary Table ST6**

**Explained variance ratios of the first two principal components in PCA calculated for each normalization factor.**

| Normalization factor | Explained variance ratio | PC1 | PC2 | PC3 | PC4 | PC5 | PC6 | PC7 |
| --- | --- | --- | --- | --- | --- | --- | --- | --- |
| 16S rRNA | Eigenvalue | 1.8851 | 1.7389 | 1.0677 | 0.8522 | 0.6502 | 0.4753 | 0.3306 |
|  | Proportion Explained | 0.2693 | 0.2484 | 0.1525 | 0.1217 | 0.0929 | 0.0679 | 0.0472 |
|  | Cumulative Proportion | 0.2693 | 0.5177 | 0.6702 | 0.7920 | 0.8849 | 0.9528 | 1.0000 |
| ENT 23S rRNA | Eigenvalue | 3.6032 | 1.1503 | 0.7398 | 0.5697 | 0.3604 | 0.3243 | 0.2523 |
|  | Proportion Explained | 0.5147 | 0.1643 | 0.1057 | 0.0814 | 0.0515 | 0.0463 | 0.0360 |
|  | Cumulative Proportion | 0.5147 | 0.6791 | 0.7848 | 0.8662 | 0.9176 | 0.9640 | 1.0000 |
| HF183 | Eigenvalue | 3.2060 | 1.2364 | 0.8826 | 0.6538 | 0.3803 | 0.3595 | 0.2812 |
|  | Proportion Explained | 0.4580 | 0.1766 | 0.1261 | 0.0934 | 0.0543 | 0.0514 | 0.0402 |
|  | Cumulative Proportion | 0.4580 | 0.6347 | 0.7607 | 0.8541 | 0.9085 | 0.9598 | 1.0000 |
| pBI143 | Eigenvalue | 3.6820 | 1.1720 | 0.7913 | 0.5417 | 0.3303 | 0.2671 | 0.2157 |
|  | Proportion Explained | 0.5260 | 0.1674 | 0.1130 | 0.0774 | 0.0472 | 0.0382 | 0.0308 |
|  | Cumulative Proportion | 0.5260 | 0.6934 | 0.8064 | 0.8838 | 0.9310 | 0.9692 | 1.0000 |
| *Carjivirus* | Eigenvalue | 3.7480 | 1.3539 | 0.8090 | 0.4203 | 0.3161 | 0.2167 | 0.1360 |
|  | Proportion Explained | 0.5354 | 0.1934 | 0.1156 | 0.0600 | 0.0452 | 0.0310 | 0.0194 |
|  | Cumulative Proportion | 0.5354 | 0.7289 | 0.8444 | 0.9045 | 0.9496 | 0.9806 | 1.0000 |

**
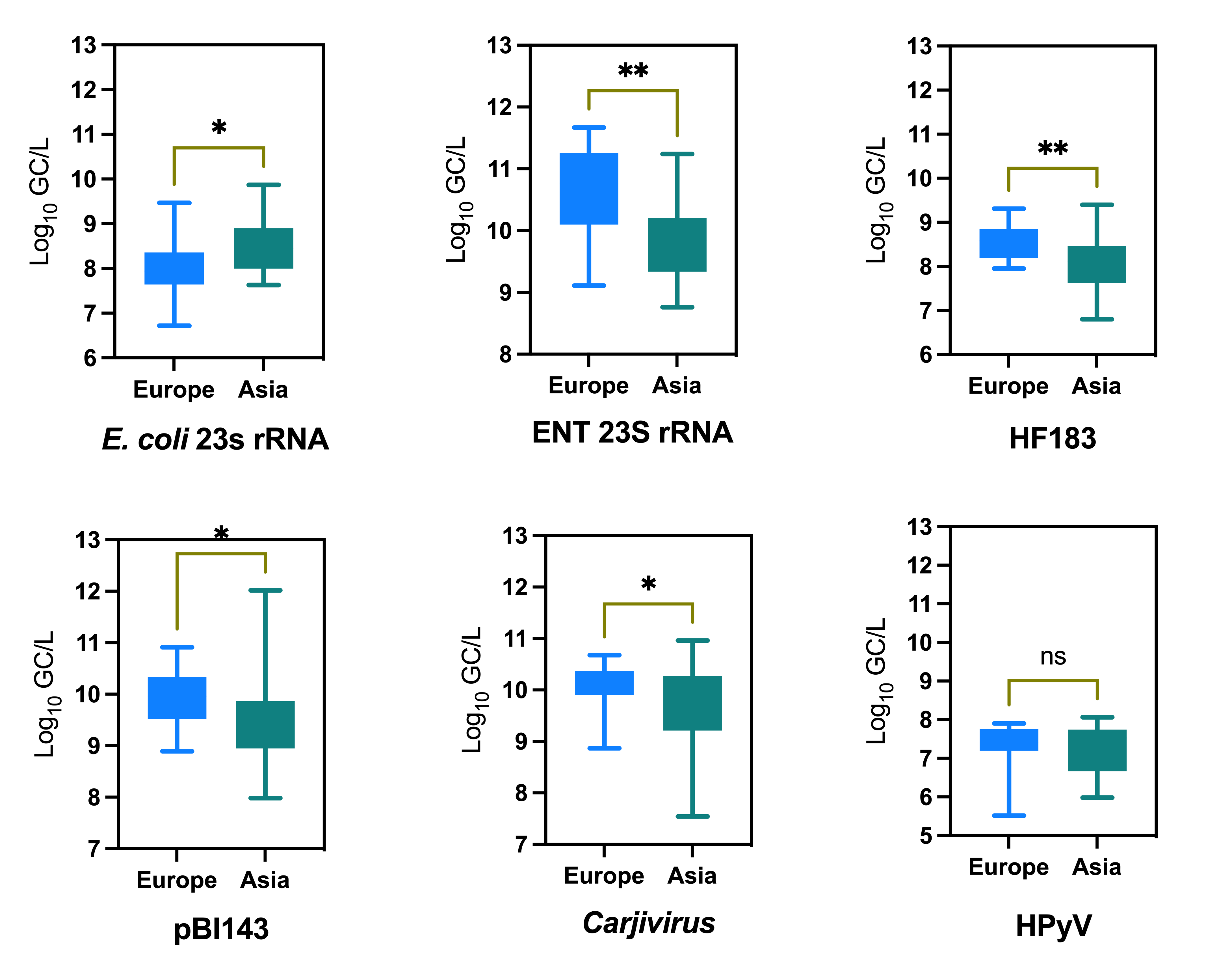
**

**Supplementary Figure SF1**

**Concentrations of six human fecal/urine markers in flights differentiated by continents (* indicates *P* <0.05, ** denotes *P* <0.01).**

**
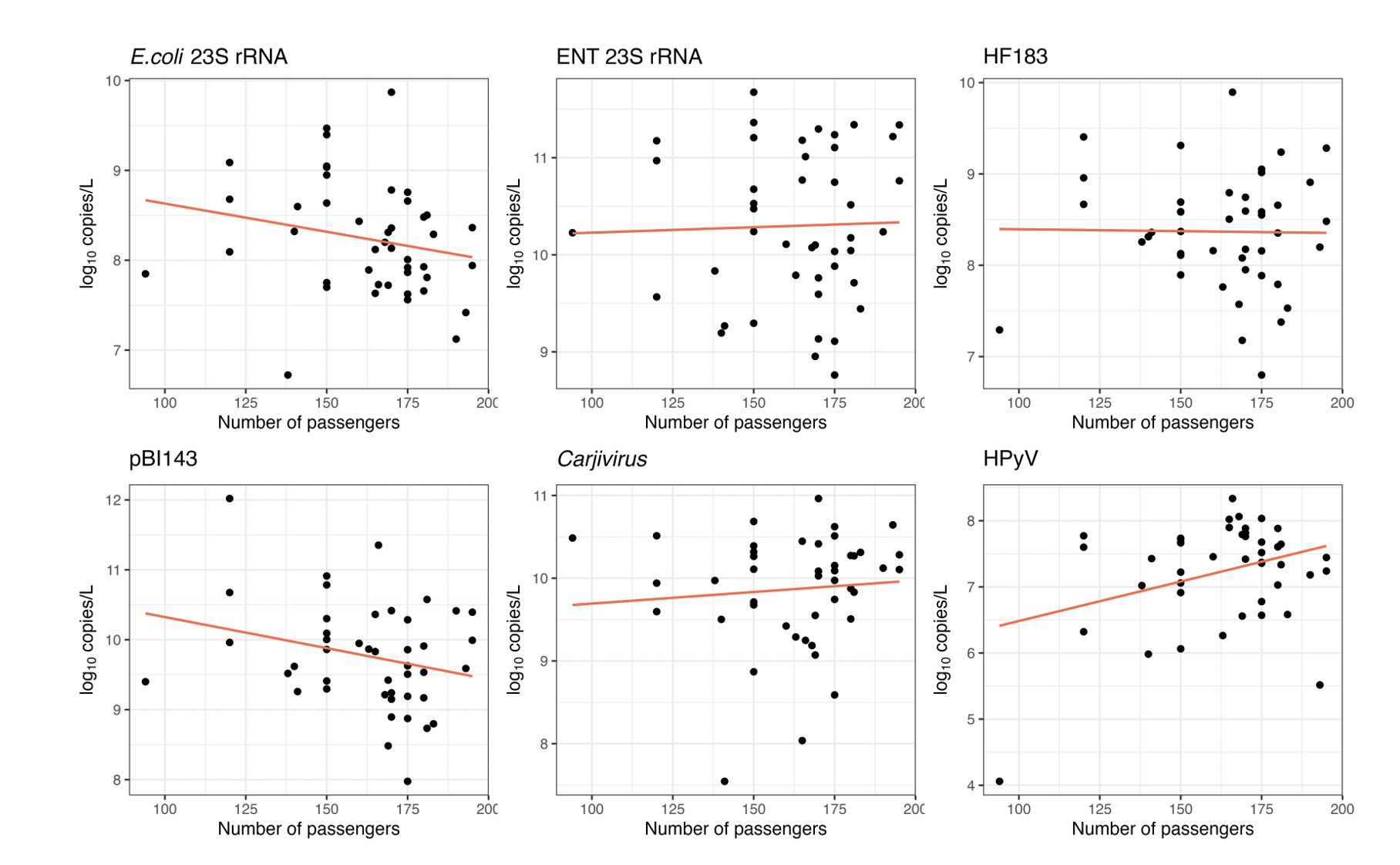
**

**Supplementary Figure SF2**

**Comparison of concentrations of six human fecal/urine markers and number of passengers onboard in flights.**

**
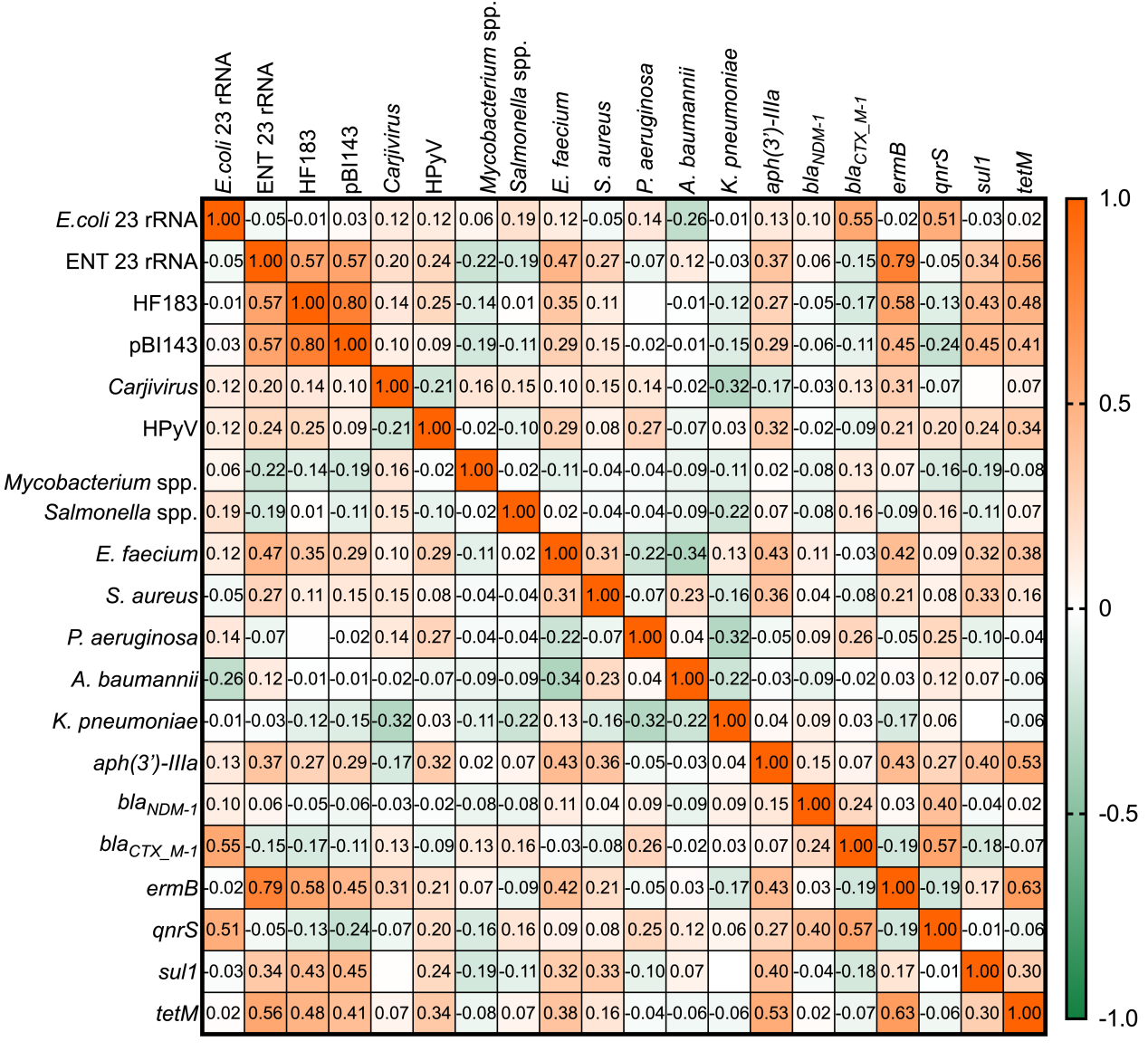
**

**Supplementary Figure SF3**

**Correlations among human fecal/urine markers, pathogens and ARGs from aggregated aircraft wastewater samples.**

**References**

1. Ritalahti, K.M., Amos, B.K., Sung, Y., Wu, Q., Koenigsberg, S.S., Löffler, F.E., 2006. Quantitative PCR targeting 16S rRNA and reductive dehalogenase genes simultaneously monitors multiple Dehalococcoides strains. Appl Environ Microbiol. 72(4):2765-74.
2. Chern, E.C., Siefring, S., Paar, J., Doolittle, M., Haugland, R.A., 2011. Comparison of quantitative PCR assays for *Escherichia coli* targeting ribosomal RNA and single copy genes. Lett Appl Microbiol. 52(3):298-306.
3. Haugland, R.A., Siefring, S.C., Wymer, L.J., Brenner, K.P., Dufour, A.P., 2005. Comparison of *Enterococcus* measurements in freshwater at two recreational beaches by quantitative polymerase chain reaction and membrane filter culture analysis. Water Res. 39:559-68.
4. Green, H.C., Haugland, R.A., Varma, M., Millen, H.T., Borchardt, M.A., Field, K.G., Walters, W.A., Knight, R., Sivaganesan, M., Kelty, C.A., Shanks, O.C., 2014. Improved HF183 quantitative real-time PCR assay for characterization of human fecal pollution in ambient surface water samples. Appl Environ Microbiol. 80(10):3086-94.
5. Fogarty, E.C., Schechter, M.S., Lolans, K., Sheahan, M.L., Veseli, I., Moore, R.M., Kiefl, E., Moody, T., Rice, P.A., Yu, M.K., Mimee, M., Chang, E.B., Ruscheweyh, H.J., Sunagawa, S., Mclellan, S.L., Willis, A.D., Comstock, L.E., Eren, A.M., 2024. A cryptic plasmid is among the most numerous genetic elements in the human gut. Cell. 187(5):1206-22.
6. Stachler, E., Kelty, C., Sivaganesan, M., Li, X., Bibby, K., Shanks, O.C., 2017. Quantitative CrAssphage PCR assays for human fecal pollution measurement. Environ Sci Technol. 51(16):9146-54.
7. McQuaig, S., Scott, T., Lukasik, J., Paul, J., Harwood, V., 2009. Quantification of human polyomaviruses JC virus and BK virus by TaqMan quantitative PCR and comparison to other water quality indicators in water and fecal samples. Appl Environ Microbiol. 75:3379-88.
8. Maheux, A.F., Bissonnette, L., Boissinot, M., Bernier, J.L., Huppé, V., Bérubé, E., Boudreau, D.K., Picard, F.J., Huletsky, A., Bergeron, M.G., 2011. Method for rapid and sensitive detection of *Enterococcus sp*. and *Enterococcus faecalis/faecium* cells in potable water samples. Water Res. 45(6):2342-54.
9. Brakstad, O.G., Aasbakk, K., Maeland, J.A., 1992. Detection of Staphylococcus aureus by polymerase chain reaction amplification of the *nuc* gene. J Clin Microbiol. 30(7):1654-60.
10. Feng, J., Cui, X., Du, B., Zhao, H., Feng, Y., Cui, J., Yan, C., Gan, L., Fan, Z., Fu, T., Xu, Z., Yu, Z., Zhang, R., Du, S., Tian, Z., Zhang, Q., Xue, G., Yuan, J., 2023. Detection and quantification of *Klebsiella pneumoniae* in fecal samples using digital droplet PCR in comparison with real-time PCR. Microbiol Spectr. 11(4): e0424922.
11. De Gregorio, E., Roscetto, E., Iula, V.D., Martinucci, M., Zarrilli, R., Di Nocera, P.P., Catania, M.R., 2015. Development of a real-time PCR assay for the rapid detection of *Acinetobacter baumannii* from whole blood samples. New Microbiol. 38(2):251-7.
12. Anuj, S.N., Whiley, D.M., Kidd, T.J., Bell, S.C., Wainwright, C.E., Nissen, M.D., Sloots, T.P., 2009. Identification of *Pseudomonas aeruginosa* by a duplex real-time polymerase chain reaction assay targeting the *ecfX* and the *gyrB* genes. Diagn Microbiol Infect Dis. 63(2):127-31.
13. Bruijnesteijn Van Coppenraet, E.S., Lindeboom, J.A., Prins, J.M., Peeters, M.F., Claas, E.C., Kuijper, E.J., 2004. Real-time PCR assay using fine-needle aspirates and tissue biopsy specimens for rapid diagnosis of *mycobacterial lymphadenitis* in children. J Clin Microbiol. 42(6):2644-50.
14. Malorny, B., Paccassoni, E., Fach, P., Bunge, C., Martin, A., Helmuth, R., 2004. Diagnostic real-time PCR for detection of *Salmonella* in food. Appl Environ Microbiol. 70(12):7046-52.
15. Paulus, G.K., Hornstra, L.M., Medema, G., 2020. International tempo-spatial study of antibiotic resistance genes across the Rhine river using newly developed multiplex qPCR assays. Sci Total Environ. 706:135733.
16. Ahammad, Z.S., Sreekrishnan, T.R., Hands, C.L., Knapp, C.W., Graham, D.W., 2014. Increased waterborne blaNDM-1 resistance gene abundances associated with seasonal human pilgrimages to the upper Ganges River. Environ. Sci. Technol. 48, 3014–3020.
17. Colomer-Lluch, M., Jofre, J., Muniesa, M., 2011. Antibiotic resistance genes in the bacteriophage DNA fraction of environmental samples. PLoS One. 6, e17549.
18. Hindiyeh, M., Smollen, G., Grossman, Z., Ram, D., Davidson, Y., Mileguir, F., Vax, M., Ben David, D., Tal, I., Rahav, G., Shamiss, A., Mendelson, E., Keller, N., 2008. Rapid detection of blaKPC carbapenemase genes by real-time PCR. J Clin Microbiol. 46(9):2879-83.
19. Chen, J., Yu, Z., Michel, F.C., Wittum, T., Morrison, M., 2007. Development and application of real-time PCR assays for quantification of erm genes conferring resistance to macrolides-lincosamides-streptogramin B in livestock manure and manure management systems. Appl. Environ. Microbiol. 73, 4407-4416.
20. Colomer-Lluch, M., Jofre, J., Muniesa, M., 2014. Quinolone resistance genes (qnrA and qnrS) in bacteriophage particles from wastewater samples and the effect of inducing agents on packaged antibiotic resistance genes. J. Antimicrob Chemother. 69, 1265-1274.
21. Ng, L.K., Martin, I., Alfa, M., Mulvey, M., 2001. Multiplex PCR for the detection of tetracycline resistant genes. Mol Cell Probes. 15, 209-215.
22. Heuer, H., Schmitt, H., Smalla, K., 2011. Antibiotic resistance gene spread due to manure application on agricultural fields. Curr Opin Microbiol. 14(3):236-43.
23. Bell, J.M., Paton, J.C., Turnidge, J., 1998. Emergence of vancomycin-resistant enterococci in Australia: phenotypic and genotypic characteristics of isolates. J Clin Microbiol. 36, 2187–2190.
